# Sildenafil citrate can improve erectile dysfunction among chronic hemodialysis patients

**DOI:** 10.4103/0971-4065.70845

**Published:** 2010-07

**Authors:** A. Ghafari, B. Farshid, A. T. Afshari, N. Sepehrvand, E. Rikhtegar, K. Ghasemi, S. Hatami

**Affiliations:** Department of Internal Medicine, Urmia University of Medical Sciences, Imam Khomeini Training Hospital, Urmia, Iran

**Keywords:** Erectile dysfunction, hemodialysis, sildenafil citrate

## Abstract

Erectile dysfunction (ED) is common among patients with end-stage renal disease (ESRD), who undergo hemodialysis (HD). The aim of this study was to evaluate the safety and effectiveness of sildenafil in male HD patients with ED. Twenty-seven HD patients were recruited for this prospective, randomized, double-blind, placebo-controlled, clinical trial study of sildenafil during a period of 1 week. Efficacy was assessed by using the International Index of Erectile Function (IIEF) before and 1 week after treatment. Baseline demographic and clinical features were similar in both the groups. There was a weak correlation between ED and duration of undergoing dialysis (*P* = 0.073). There was significant relationship between sildenafil usage and improvement in erectile function (*P* < 0.0001). Placebo improved significantly the erectile function (*P* = 0.016), perhaps by psychological way. However, sildenafil had a more significant effect than placebo in increasing IIEF score among HD patients (*P* = 0.00 compared to 0.016). Sildenafil is effective and safe for treating ED among HD patients.

## Introduction

Erectile dysfunction (ED) is the inability to achieve and/or maintain an erection for a satisfactory sexual performance.[1] Advanced chronic kidney disease (CKD) is associated with impaired spermatogenesis and testicular damage.[2] The incidence of ED is estimated to be between 50 and 70% depending on the stage of renal failure.[3] In fact sexual dysfunction (SD) is composed of both physiological and psychological factors.[4] The ED improves after successful kidney transplantation.[5]

Dialysis improves most symptoms of end-stage renal disease (ESRD), yet many patients continue to experience SD during the dialysis treatment.[6] A revolution occurred in the treatment of ED with the introduction of sildenafil in the last decade.[7] The use of sildenafil seems to be more valuable in young patients with ED which appeared after long dialysis duration.[8] The aim of this study was to evaluate the safety and effectiveness of sildenafil in the treatment of ED in Iranian male hemodialysis (HD) patients.

## Materials and Methods

We designed a double-blind, randomized, placebo-controlled clinical trial study. This study was approved by the Review board of Urmia University of Medical Sciences.

The patients were informed sufficiently about how to use tablets, the probable complications, the symptoms of improvement, and also about international index of erectile function (IIEF) questionnaire, and after that an informed consent was obtained from each research subject.

The self-administered IIEF, a 15-question validated measure of ED, was used to evaluate ED and the treatment response. The IIEF allows its identification and its stratification into several grades according to the scores obtained in the erectile function domain: absence of ED (score ≥21), mild ED (score 16–20), moderate ED (score 11–15), and severe ED (score 5–10).

Patients on chronic HD who had received treatment for at least 6 months, having had a stable relationship with a female sexual partner and with an IIEF score lower than 20 or a subjective complaint of ED were included in our study.

The patients without sexual activity were excluded because they did not perceive their deficiency as a problem and considered this situation inherent to the aging process.[9]

Patients older than 70 years with penile anatomic abnormalities, cirrhosis, diabetes, angina, severe anemia (hematocrit lower than 21%), and those who were on nitrate treatment or had a recent history of stroke or myocardial infarction, under treatment for ED, or patients with systolic BP higher than 170 or lower than 90 were excluded. We did not include patients who underwent continuous ambulatory peritoneal dialysis (CAPD) because it is not common in our region and the number of patients under CAPD who met the inclusion and exclusion criteria was not enough.

The patients were divided randomly into two categories: intervention and control groups. Two groups were matched according to age and baseline IIEF score. Groups were not matched for other comorbidities due to chronic renal disease. We changed into powder every 50-mg sildenafil citrate tablet made by Pfizer, New York City, NY, USA and filled it in capsules. Placebo was prepared using identical capsules. We also prepared a randomization list, and patients in each group received a sealed box containing the capsules. None of the authors or patients had access to the drug codes until all patient evaluations were finished. We delivered three capsules including sildenafil to the intervention group and three similar capsules including placebo to the control group for each day until 1 week. We encouraged them to take one capsule after the dialysis session and the second capsule 1 hour before the sexual intercourse. If they experienced no improvement in their sexual function, they could take two capsules (100 mg) before their next intercourse. All the patients underwent HD regularly three times a week. So, every participant in the intervention group received 50–150 mg/day sildenafil depending on the satisfaction of his sexual function in the last night and also concurrency with the day of dialysis. The efficacy of sildenafil was assessed by re-administering the IIEF questionnaire after a week of therapy. The safety of sildenafil was assessed by inquiry about the side effects of sildenafil administration (including nausea, palpitation, flushing, and chest pain).

One-sample Kolmogorov-Smirnov test was used for evaluating data distribution. The results of the placebo and sildenafil groups were compared using the t-test and Mann-Whitney *U* test for parametric and non-parametric data, respectively. To simplify the presentation of results, only mean (SD) and paired and un-paired *t*-test results are shown.

## Results

A total of 27 patients who underwent chronic HD were enrolled in our study and were classified into two groups: the first one was the control group which consisted of 13 patients receiving placebo and the second one was the intervention group which consisted of 14 patients receiving sildenafil.

Mean patient age was 46.5 ± 9.93 years (between 26 and 60 years). According to the influence of increasing age on ED, intervention and control groups were age-matched. We classified patients by age into three categories: lower than 40 years, 41–50 years, and 51–60 years. There was no significant relationship between age groups and ED in this study. The prevalence of ED was 60.3%. A progressive increase with respect to the age was reported. In patients younger than 50 years this prevalence reached 31.4%, and in patients older than 50 years this prevalence reached 68.6%. The time of HD varied between 6 months and 14 years. There was a weak correlation between ED and time of dialysis (*P* = 0.073) [Table 1].

**Table 1:** The effect of sildenafil compared to placebo in increasing IIEF score

	Placebo	Sildenafil
Age	48.77±8.26	44.5±11.17
First IIEF score	13.08±6.82	11.07±5.59
Second IIEF score	14±7.49	16.86±5.78
*P* value in efficiency	0.016	<0.001
Increased score	0.92±1.18	5.79±2.45

By a *t*-independent test, the two groups were evaluated and matched for their first IIEF score and found to have no significant difference. Mean score in intervention group before the intervention was 11.07 ± 5.59 and mean score in that group after implementing intervention was 16.94 ± 5.78. There was a significant relationship between sildenafil usage and ED (*P* = 0.00).

Also in the control group, mean score was 13.08 ± 6.82 before placebo and 14 ± 7.49 after placebo. So, using a placebo had improved significantly the erectile function (*P* = 0.016). According to the performed t-test, sildenafil has more effects than the placebo in improving the erectile function (*P* < 0.0001).

Among the 14 patients in intervention group, 5 responded to 100 mg doses of sildenafil and 9 were treated by a single 50-mg sildenafil tablet daily. We applied Chi-square test to evaluate the correlation between the dose of sildenafil and increased score of IIEF, but no relationship was found (*P* = 0.13).

Administration of sildenafil had improved all the domains of the questionnaire, but the placebo only affected positively the third domain, which is penetration ability.

Safety was assessed by inquiry about the side effects of sildenafil and patients reported no side effect because of using sildenafil or placebo, in intervention or control groups [Tables 2 and 3].

**Table 2:** Questionnaire’s different domains in evaluating ED

Questionnaire	Domain
Q1	Erection frequency
Q2	Erection firmness
Q3	Penetration ability
Q4	Maintenance frequency
Q5	Maintenance ability to reach orgasm

ED = erectile dysfunction

**Table 3:** The effect of sildenafil on IIEF scores compared to placebo

	IIEF	First score	Second score	*P* value
Placebo	Q1	2.77	3.00	0.082
	Q2	2.31	2.46	0.165
	Q3	2.54	2.92	0.018
	Q4	2.62	2.77	0.165
	Q5	2.58	2.58	-
	Sum	13.08	14	0.016
Sildenafil	Q1	2.29	2.93	<0.001
	Q2	2.29	3.86	<0.001
	Q3	2.21	3.93	0.001
	Q4	2.14	3.29	<0.001
	Q5	2.14	2.93	<0.001
	Sum	11.07	16.94	<0.001

IIEF = international index of erectile function

## Discussion

In this study, administration of sildenafil had improved all the domains of the erectile function. So, the study demonstrated that sildenafil could be a good option to improve sexual life of ESRD patients.

In the study of Seibel *et al*, sildenafil was associated with improvement in the score of all questions and domains of the IIEF, except those related to sexual desire.[10] Treatment with sildenafil was found as a valid option with an effective response in renal transplant recipients too.[11]

In this study, placebo also had improved the erectile function. It influenced the penetration ability. It could be justified by the psychological effects of administering placebo, especially considering the short term of the study, which can maximize the placebo effect. This improvement by placebo was also shown by Seibel *et al*, in Brazil, in which 9.5% of patients receiving placebo had experienced improvement in their erectile performance.[10]

ED has a major negative impact on the quality of life (QOL) and family relations. Its treatment is associated with improvement of psychogenic factors.[12] The presence of depressive symptoms, highly prevalent in HD patients, is an independent factor of SD in male HD patients. In a comprehensive approach to the management of SD, a thorough evaluation of psychological depression should be included.[4]

The placebo may have effect on the important psychological factors such as depression in HD patients. However, sildenafil had a more significant effect than placebo in increasing IIEF score among HD patients (*P* = 0.00 compared to 0.016).

ED in patients who developed ESRD due to diabetes mellitus probably requires higher doses of sildenafil than patients with ED, for example, due to hypertension. In our study, there were no significant relationship between the doses of sildenafil and increased score of IIEF. We did not differentiate between different underlying diseases, so perhaps they had a confounding effect on the results.

Indeed, QOL measures are subjective or objective, functional or satisfaction-based.[13] In this study, IIEF is a subjective, satisfaction-based measure. So, recall bias might have occurred in recalling about their sexual function. Also, ED is a sensitive topic and many patients do not spontaneously discuss it with their physician.[12]

In a study by Sahin *et al*, side effects of sildenafil were reported to be nausea, palpitation, flushing, and angina.[14] In this study no side effects were reported on using sildenafil or placebo. It could be related to very limited and precise inclusion and exclusion criteria of this study, or due to the short term of study. We excluded any kind of suspected history for angina or previous history of prescribing nitrate, which could inhibit probable adverse effects. As demonstrated in this study and in a similar study by Ji *et al*, sildenafil is effective and safe.[15]

Physicians and other health professionals need to pay attention to the erection problems in HD patients in order to provide directions for an adequate medical treatment.[16] As mentioned in the literature, if the oral drug fails to improve erectile function, we can choose intracavernosal injection and penile prosthesis implantation.[15]

The challenge for the next decade will be to continue to devise interventions that meaningfully increase the QOL of patients with CKD at all stages.[13] ED is one factor which influences the QOL in these patients. Evaluations for ED should be included in the routine assessment of HD patients.[12,17]

## Limitations

Possible limitations which may cause fallacy in the results of our study are small sample size (27 subjects compared to 34 subjects calculated by sample size formula for parallel clinical trial studies), very short duration of follow up, and potential changes in the bioavailability of sildenafil due to physical modification into powder.

## Conclusion

The prevalence of ED is high (60%) in patients with ESRD who underwent HD. Sildenafil citrate is effective and safe for treating ED among HD patients.

## References

[1] Russo D, Musone D, Alteri V, Cindolo L, Lanzillo B, Federico S (2004). Erectile dysfunction in kidney transplanted patients: Efficacy of sildenafil. J Nephrol.

[2] Lessan-Pezeshki M, Ghazizadeh S (2008). Sexual and reproductive function in end-stage renal disease and effect of kidney transplantation. Asian J Androl.

[3] Kleinclauss F, Kleinclauss C, Bittard H (2005). Erectile dysfunction in renal failure patients and renal transplant recipients. Prog Urol.

[4] Peng YS, Chiang CK, Hung KY, Chiang SS, Lu CS, Yang CS (2007). The association of higher depressive symptoms and sexual dysfunction in male haemodialysis patients. Nephrol Dial Transplant.

[5] Mehrsai A, Mousavi S, Nikoobakht M, Khanlarpoor T, Shekarpour L, Pourmand G (2006). Improvement of erectile dysfunction after kidney transplantation: The role of the associated factors. Urol J.

[6] Soykan A, Boztas H, Kutlay S, Ince E, Nergizoglu G, Dileköz AY (2005). Do sexual dysfunctions get better during dialysis? Results of a six-month prospective follow-up study from Turkey. Int J Impot Res.

[7] Sam R, Patel P (2006). Sildenafil in dialysis patients. Int J Artif Organs.

[8] Zamd M, Gharbi MB, Ramdani B, Zaid D (2005). Sexual dysfunction in male patients undergoing hemodialysis in morocco. Saudi J Kidney Dis Transpl.

[9] Martín-Díaz F, Reig-Ferrer A, Ferrer-Cascales R (2006). Sexual function and quality of life in hemodialysis male patients. Nefrologia.

[10] Seibel I, Poli De Figueiredo CE, Telöken C, Moraes JF (2002). Efficacy of oral sildenafil in hemodialysis patients with erectile dysfunction. J Am Soc Nephrol.

[11] Sharma RK, Prasad N, Gupta A, Kapoor R (2006). Treatment of erectile dysfunction with sildenafil citrate in renal allograft recipients: A randomized, double-blind, placebo-controlled, crossover trial. Am J Kidney Dis.

[12] Pourmand G, Emamzadeh A, Moosavi S, Mehrsai A, Taherimahmoudi M, Nikoobakht M (2007). Does renal transplantation improve erectile dysfunction in hemodialysed patients? What is the role of associated factors?. Transplant Proc.

[13] Kimmel PL, Patel SS (2006). Quality of life in patients with chronic kidney disease: Focus on end-stage renal disease treated with hemodialysis. Semin Nephrol.

[14] Sahin Y, Aygün C, Peskircioglu CL, Kut A, Tekin MI, Ozdemir FN (2004). Efficacy and safety of sildenafil citrate in hemodialysis patients. Transplant Proc.

[15] Ji ZG, Tian Y, Chen LS (2007). Influential factors and therapeutic options for erectile dysfunction in allograft renal transplantation recipients. Zhonghua Nan Ke Xue.

[16] Messina LE, Claro JA, Nardozza A, Andrade E, Ortiz V, Srougi M (2007). Erectile dysfunction in patients with chronic renal failure. Int Braz J Urol.

[17] Arslan D, Aslan G, Sifil A, Cavdar C, Celebi I, Gamsari T (2002). Sexual dysfunction in male patients on hemodialysis: Assessment with the International Index of Erectile Function (IIEF). Int J Impot Res.

